# 2362. SARS-CoV-2 Omicron Variants Associated with Less Severe Disease in Hospitalized Patients Irrespective of Baseline Risk Factors and Immunity

**DOI:** 10.1093/ofid/ofad500.1983

**Published:** 2023-11-27

**Authors:** Austin Lee, Eric Kawaguchi, Christina Vu, Brianna Ray, Joy Suh, Vanya Vojvodic, Scotti Smith, Cesar Torres, Chrysovalantis Stafylis, Noah Wald-Dickler, Paul Holtom, Jeffrey Klausner, Saahir Khan

**Affiliations:** Department of Medical Education, Keck School of Medicine, University of Southern California, Los Angeles, California, USA, Los Angeles, California; Department of Population and Public Health Sciences, Keck School of Medicine, University of Southern California, Los Angeles, California, USA, Los Angeles, California; DAPHealth, Palm Springs, CA, Los Angeles, California; Legacy Medical Group, Portland, OR, Los Angeles, California; Department of Medical Education, Keck School of Medicine, University of Southern California, Los Angeles, California, USA, Los Angeles, California; Department of Medical Education, Keck School of Medicine, University of Southern California, Los Angeles, California, USA, Los Angeles, California; Department of Medical Education, Keck School of Medicine, University of Southern California, Los Angeles, California, USA, Los Angeles, California; Department of Medical Education, Keck School of Medicine, University of Southern California, Los Angeles, California, USA, Los Angeles, California; Department of Population and Public Health Sciences, Keck School of Medicine, University of Southern California, Los Angeles, California, USA, Los Angeles, California; LA County + University of Southern California Medical Center; LAC+USC Medical Center, Los Angeles, California; Keck School of Medicine of USC, Los Angeles, California; Department of Medicine, Keck School of Medicine, University of Southern California, Los Angeles, California, USA, Los Angeles, California

## Abstract

**Background:**

The Omicron variant of SARS-CoV-2 saw the highest incidence of cases during the COVID-19 pandemic, yet these cases had less severe outcomes compared to earlier variants. This could be due to increased immunity from vaccination, infection of healthier patients, increased identification of less severe cases, and decreased viral pathogenicity. We examine the relative contribution of these factors to outcomes among hospitalized COVID-19 patients by comparing a cohort from the early pandemic to a cohort from the Omicron wave, subdivided by baseline immunity.

**Methods:**

We retrospectively reviewed electronic medical records of 456 patients hospitalized for COVID during the early pandemic (03/2020 - 05/2020) and 453 patients hospitalized for COVID during the Omicron wave (12/2021 - 01/2022) at a public safety net hospital in Los Angeles, CA. We recorded demographics, clinical data, infection and vaccination history, and hospital outcomes including severe disease, defined as need for supplemental oxygen.

**Results:**

Patients hospitalized during the early pandemic were more likely to have severe disease than those during the Omicron wave (75.9% vs 35.1%, p < 0.001). Within the Omicron cohort, “non-immune” patients without vaccination or prior infection had more severe disease than “immune” patients with prior vaccination or infection (37.8% vs 33.3%, p < 0.001). Patients hospitalized during the early pandemic were older with more medical comorbidities than during the Omicron wave; within the Omicron cohort, immune patients were older with more medical comorbidities than non-immune patients. After controlling for age and medical comorbidities via logistic regression, non-immune Omicron patients were less likely to have severe disease than early pandemic patients (OR = 0.25 [0.17-0.38]); immune Omicron patients had even lower rates of severe disease compared to early pandemic patients (OR = 0.15 [0.10-0.23]).

Characteristics of Patient Cohorts divided by Pandemic Period and Immunity Status

**Figure:**
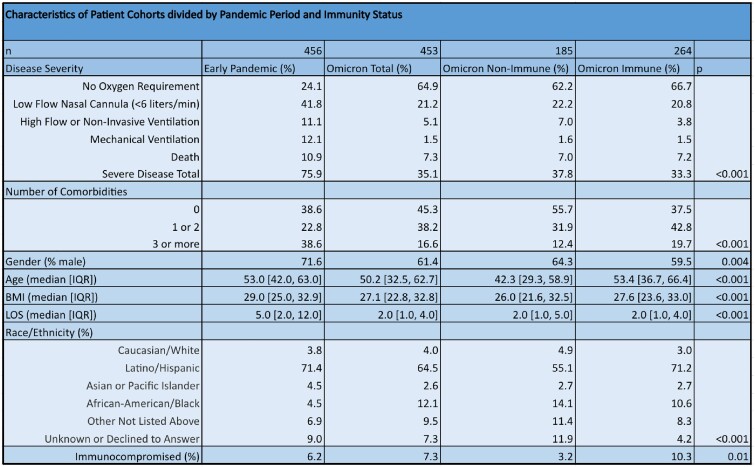

Risk Factors associated with Severe Disease by Logistic Regression

**Figure:**
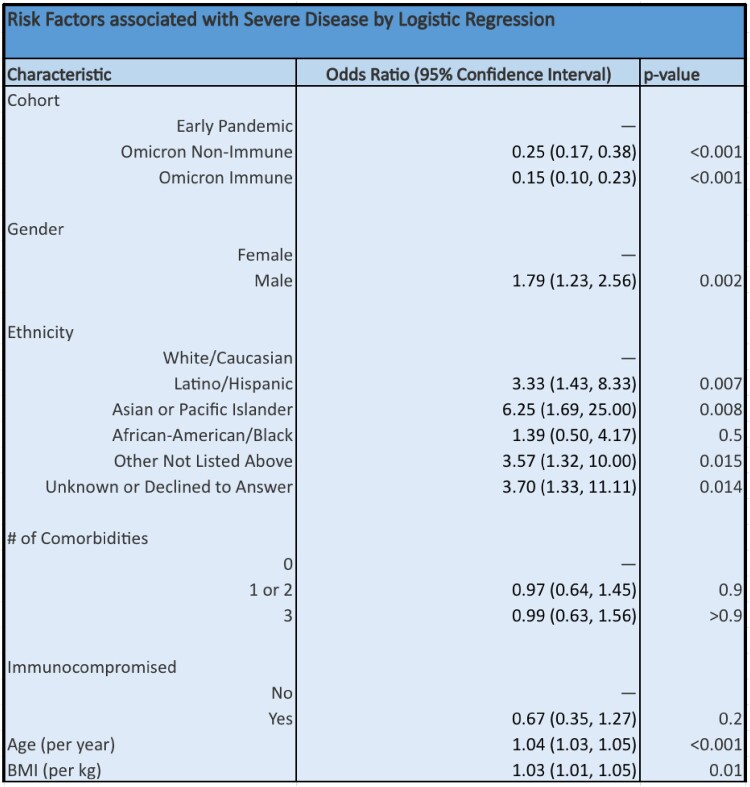

**Conclusion:**

We conclude that the Omicron variant of SARS-CoV-2 produced milder disease than earlier variants irrespective of baseline risk factors and immunity, but vaccination or prior infection further reduced disease severity in the Omicron cohort. The likely presence of undetected prior infection in the Omicron cohort would weaken the first but strengthen the second conclusion.

**Disclosures:**

**All Authors**: No reported disclosures

